# Benefits of Psychological Detachment From Work: Does Autonomous Work Motivation Play a Role?

**DOI:** 10.3389/fpsyg.2020.00824

**Published:** 2020-04-30

**Authors:** Anja Hagen Olafsen, Marte Bentzen

**Affiliations:** ^1^School of Business, University of South-Eastern Norway, Kongsberg, Norway; ^2^Department of Physical Education, Norwegian School of Sport Sciences, Oslo, Norway

**Keywords:** job recovery, psychological detachment, autonomous work motivation, self-determination theory, well-being, work functioning

## Abstract

Research has shown that psychological detachment from work during non-work time is beneficial for various aspects of employee well-being and job performance. However, it is uncertain whether psychological detachment is equally important to all. The purpose of the current study was to examine whether psychological detachment is less important for employees who experience autonomous motivation for their job. The study was conducted in two different samples of knowledge workers in Norway. Latent profile analysis was used to identify different subgroups within the samples. In addition, the BCH method was used to examine possible differences between the profiles on distal outcomes. In both samples, two distinct profiles were found: “Lower involvement employees” (Low-IE; higher detachment and lower autonomous regulation) and “Higher involvement employees” (High-IE; lower detachment and higher autonomous regulation). The results revealed that the High-IE profile was significantly higher in positive affect, life satisfaction, work effort, and work quality, while significantly lower on emotional exhaustion compared with Low-IE. These results indicate that being low in detachment from work does not seem to be detrimental when combined with high levels of autonomous motivation. The study contributes to knowledge about the benefits of unwinding from work for employees with different motivational profiles.

## Introduction

Recovery from work refers to the process of reducing or eliminating physical and psychological strain symptoms that have been caused by work (Sonnentag and Fritz, 2007). Job recovery is increasingly important in today’s work life where it can be argued that the lines between work and leisure time often times can be blurred. This is rooted in the change toward a knowledge society, in which there is an increased proportion of knowledge workers with intellectually demanding jobs who might find it difficult to detach from work, even though they have physically left their workplace. Moreover, the rapid development of technology has enabled us to be digitally present and available 24/7. These developments also provide opportunities for more flexible working hours and opportunities to work away from the office. As a result, today’s work life might make it difficult to mentally switch off from work after working hours in order to recover and focus solely on other activities.

Over the past decade, a research stream has examined the importance of recovery processes for employee well-being (for a review, see Sonnentag et al., 2017). In this literature, the role of psychological detachment as a core dimension of job recovery has received considerable attention, and various studies have elucidated the numerous benefits of psychological detachment from work related to employees’ psychological well-being as well as their work performance. For instance, psychological detachment has been shown to decrease exhaustion, psychological strain, depressive symptoms, health complaints, and sleep problems, while increasing life satisfaction (Sonnentag and Fritz, 2007; Siltaloppi et al., 2009; Sonnentag et al., 2010). Furthermore, some studies have indicated that psychological detachment is positively linked to work performance (Binnewies et al., 2010). These results are shown using both between-person and within-person study designs indicating that psychological detachment vary both between and within individuals (Sonnentag and Fritz, 2015).

While the literature on psychological detachment has identified important benefits of unwinding from work, the question of whether psychological detachment is equally beneficial to all employees remains unanswered (Sonnentag, 2018). The purpose of the current study is to examine whether psychological detachment has the same positive benefits for all employees when their quality of work motivation is taken into account. To examine this question, we draw on self-determination theory, which distinguishes between different qualities of motivation. Numerous studies in recent decades provide support for the benefits of autonomous work motivation for employee well-being and work functioning (for a review, see Deci et al., 2017). Thus, in this study, we examine whether the benefits of psychological detachment are similar for employees with various levels of autonomous work motivation.

### Job Recovery – The Importance of Psychological Detachment

Theories on recovery experiences include frameworks such as the effort–recovery model (Meijman and Mulder, 1998) and the conservation of resources theory (Hobfoll, 1998). The effort–recovery model assumes that the psychological and physiological systems that have been activated during the working day will return to, and stabilize on, what Meijman and Mulder (1998) refer to as the baseline value. If conditions are optimal, the employee will be able to recover by taking a break from work. However, it may happen that an employee is not able to disconnect from work, and thus the recovery process will be incomplete. As a result, the employee may still be tired after the previous working day when at work the next day, and must therefore make more effort to perform well enough in their job (Geurts and Sonnentag, 2006). This can build up stress reactions over time and result in chronic and long-term health problems such as fatigue, exertion, sleep problems, and other psychosomatic disorders (Meijman and Mulder, 1998). In addition, it is important to restore resources for the employee to perform optimally in his/her job (Fritz and Sonnentag, 2006).

Conservation of resources theory assumes that people strive to preserve, protect and build personal, social, and material resources (Hobfoll et al., 2018). It is believed that a potential or actual loss of such valuable resources is perceived as threatening by the individual (Hobfoll, 1998). As a result, stress occurs, which is detrimental to health and well-being. To prevent the stress reactions from continuing over time, individuals must acquire new resources and restore endangered or lost resources (Sonnentag and Fritz, 2007). One way of doing this is to engage in recreational activities that can add new energy, and thus achieve psychological relaxation from the job (Siltaloppi et al., 2009).

Taken together, the effort-recovery model and the conservation of resources theory suggest two complementary processes by which recovery occurs. First, it is important to refrain from work demands and to avoid activities that call upon the same functional systems or internal resources as those required at work. Second, gaining new internal resources such as energy, self-efficacy, or positive mood will additionally help to restore threatened resources (Sonnentag and Fritz, 2007). Based on these considerations, Sonnentag and Fritz (2007) proposed four strategies in the recovery process: psychological detachment from work, relaxation-oriented strategies, mastery-oriented strategies, and control.

Of the four recovery strategies, the one that has received by far the most attention in the literature to date is that of psychological detachment. By disconnecting mentally from work, the employee is freed from work-related demands, and psychological detachment is therefore of crucial importance for the recovery process (Nohe et al., 2014). Employees who remain mentally present at work after the working day are thus not fully benefiting from their spare time (Sonnentag and Bayer, 2005). However, to achieve psychological detachment, it is necessary to avoid activities related to work, such as reading and answering job-related e-mails, because such activities prevent psychological relaxation (Siltaloppi et al., 2009). It has therefore been argued that psychological detachment includes both resisting engaging in job-related activities *and* thinking about job-related issues during leisure time (Sonnentag and Bayer, 2005; Sonnentag and Fritz, 2007; Sonnentag et al., 2010).

Sonnentag (2010) proposed the stressor-detachment model that emphasizes psychological detachment as an important factor to consider in the stressor–strain process. In this model, psychological detachment is displayed both as a mediator and as a moderator in the relation between stressors and strain. That is, job stressors can result in lower psychological detachment that, in turn, directly influences an employee’s level of strain and well-being, but also function as a buffer for the negative effects of job stressors on strain and poor well-being. Research has particularly used this model to examine how psychological detachment from work relates to people’s mood states and well-being. In particular, recent meta-analyses have revealed psychological detachment as an important predictor of positive mood states and well-being, whereby psychological detachment has been linked to lower levels of burnout, fatigue, and physical discomfort, and higher levels of vigor, sleep quality, and overall well-being (Wendsche and Lohmann-Haislah, 2017; Bennett et al., 2018; Headrick et al., 2019; Steed et al., 2019). In addition, while most studies have emphasized the implications of psychological detachment on employee well-being, some have also highlighted implications for employee performance. However, the literature is not consistent when it comes to this relation and meta-analytic findings suggest both a reduction in performance when employees are higher in psychological detachment (Headrick et al., 2019) as well as a small positive association between psychological detachment from work and work performance (Wendsche and Lohmann-Haislah, 2017).

Despite these positive consequences of psychological detachment from work for most work-related outcomes, Sonnentag et al. (2010) refer to several studies indicating that this is not necessarily always the case as it may depend on the content of job-related thoughts during non-work time. For instance, thinking positively about one’s work (Meier et al., 2016) or engaging in problem solving (Querstret and Cropley, 2012) may have positive associations with well-being. Similarly, positive reflections on work during weekends and holidays might increase the employee’s well-being when back at work (Sonnentag et al., 2010). In an extension of the stressor-detachment model, Sonnentag and Fritz (2015) indeed proposed moderators to this model as the associations between the model’s core constructs might differ between individuals and situations. In particular, in this extended stressor-detachment model, the content of job-related thoughts are presented as a potential moderator in the relation between psychological detachment and well-being. Following this, Sonnentag (2018) speculates about whether people seeing their work as a calling might reduce their need for recovery, while at the same time making it difficult to achieve recovery. However, Sonnentag and Fritz (2015) states that while such moderators are warranted based on conceptual arguments, empirical research on such moderator effects is scarce. We now discuss this question in light of the framework of self-determination theory.

### Self-Determination Theory

Self-determination theory (Deci and Ryan, 2000; Ryan and Deci, 2017) is a macro theory of human motivation that has been applied across empirical domains, and is today one of the most prominent theories in explaining work motivation, work functioning, and organizational behavior in general (Deci et al., 2017). Self-determination theory emphasizes the importance of the quality of people’s motivation, which is seen as much more important than the amount. A distinction is made between extrinsic and intrinsic motivation, where extrinsic motivation refers to engaging in an activity for instrumental reasons such as gaining a reward or avoiding punishment, while intrinsic motivation refers to engaging in an activity due to the experience of wanting to engage in it due to interest and enjoyment (Ryan and Deci, 2017). However, it is acknowledged that extrinsic motivation can vary in its degree of autonomy based on its degree of internalization—-the process of how values, behaviors, and beliefs are taken in by individuals and made their own—-and has distinct consequences. Hence, a more important distinction than that between intrinsic and extrinsic motivation is made between controlled and autonomous motivation. Controlled motivation refers to acting with a sense of pressure, while autonomous motivation refers to acting with a sense of volition and the experience of choice (Ryan and Deci, 2017).

Four types of extrinsic motivation regulations are identified in the framework. *External regulation* involves performing an activity to get a reward or to avoid punishment. *Introjection* is based on guilt feeling and the experience of coercion; the person would rather not engage in the activity, but feels pressured into doing so to feel well or to avoid a bad conscience. *Identification* means engaging in an activity because it is perceived as important in relation to personal goals, values, and identity; the person chooses to engage in the activity because it is considered important and useful by the person. The final form of external motivation is *integration*, which involves relating to the value of an activity in such a way that the activity becomes an integral part of oneself. Controlled motivation represents both external and introjected regulation, while autonomous motivation represents both the two most autonomous external regulations, namely identified and integrated regulations, and intrinsic motivation (Ryan and Deci, 2017).

Numerous studies within the work domain, have shown that autonomous work motivation is linked to a variety of positive outcomes related to employee well-being and work functioning. Contrary, controlled work motivation has not only been linked to less positive outcomes and can also be detrimental to health and functioning in the work life (for a review, see Deci et al., 2017). With this in mind, the present study explores the associations between psychological detachment, autonomous work motivation, and workers’ well-being and functioning.

Both psychological detachment and quality of work motivation play a part in explaining occupational health outcomes. Given the profound positive implications of autonomous work motivation on work-related psychological health and work functioning, it seems plausible that people who are high in autonomous work motivation do not necessarily experience the same benefits of psychologically detaching from work. That is, when these employees think about their work, it is likely to be in a positive manner as they experience meaning, value, and/or interest in relation to work. As such, psychological detachment might not have the same effect for their work-related psychological health and work functioning. Indeed, results from Casper et al. (2019) suggest that positive and negative work reflections during leisure time differ in their relation to energetic well-being, where persons with positive reflections experience the highest well-being and persons with negative reflections experience the lowest well-being. Such results might indicate that the effect of psychological detachment based on the employees’ underlying work affect, which should have clear relations to also type of motivation toward the job.

### The Present Study

In the last decade, research using a person-centered approach has been relatively established within organizational psychology (Morin et al., 2018). The advantage of person-centered approaches is that they identify and classify smaller and more homogenous subgroups within the total sample, were individuals belonging to each of the subgroups are similar to one-another based on the values of one or several variables (Berlin et al., 2014). This approach further allows for an exploration of how different profiles are related to a set of associated variables/outcomes.

Within previous organizational research, employers’ motivational profiles have been explored in several studies (e.g., Moran et al., 2012; Van den Berghe et al., 2014; Howard et al., 2016). In all of these studies, latent profiles have been explored by only looking at the different motivational regulations. In the recovery literature, profile studies have typically studied psychological detachment together with the other three recovery experiences to explore distinct recovery profiles and their antecedents and outcomes (Bennett et al., 2016; Gabriel et al., 2019; Chawla et al., 2020). In the present study, by using a person-centered approach, we extend the current literature by exploring whether there are unique subgroups (i.e., profiles) of workers based on their level of self-reported psychological detachment from a workday to the next and their autonomous work motivation. This approach makes it possible to model alternative combinations of detachment and autonomous work motivation among employees, in contrast to a variable-centered approach where psychological detachment and autonomous work regulations would be examined in isolation (Wang and Hanges, 2011). Further, by capturing heterogeneity that would have gone unobserved in a variable-centered approach (Wang and Hanges, 2011; Bennett et al., 2016), this approach allows for detection of subgroups of these combinations that exist in the work context that may relate distinctively to work outcomes, which may help explain ambiguous results in past research. In sum, this person-centered approach enables the examination of whether psychological detachment is equally beneficial for all employees, taking autonomous work motivation into account, in line with the extended stressor-detachment model (Sonnentag and Fritz, 2015).

As this is the first study to, our knowledge, combining these variables in LPA, we have an exploratory approach, both in terms of the number of profiles and how the combination of levels of included variables within the profiles will vary. Second, if subgroups are detected, the study will further investigate if there are potential differences between subgroups on self-reported well-being (i.e., affect, emotional exhaustion, somatic symptom burden, life satisfaction, vigor, work–home interference, and sleep quality) and work functioning (i.e., work effort and work quality) in two separate studies.

## Materials and Methods

### Study 1 Participants and Procedures

Participants were 239 employees (58.6% women, 41.4% men) from three knowledge-intensive firms in Norway, with an age range of 25–69 years (*M* = 43.65, *SD* = 11.02). Of the respondents, 78.2% had higher education (bachelor’s degree or higher) and the sample comprised employees from both the public (57.7%) and private (42.3%) sectors. The data were collected using an electronic survey distributed either by an e-mail invitation (company 1 and 3) or through intranet (company 2).

### Study 1 Measures

#### Psychological Detachment

Psychological detachment from work was measured by the Recovery Experience Questionnaire (Sonnentag and Fritz, 2007). Respondents were asked to consider how the claims corresponded to how they generally experience disconnecting from work between two working days. The four items (e.g., “I forget about work”) were reported on a scale ranging from 1 (completely disagree) to 5 (completely agree).

#### Work Motivation

The autonomous work motivation of the respondents was assessed using the Multidimensional Work Motivation Scale (Gagné et al., 2015). Two of the described autonomous regulations/motivations are represented in this scale, where the participants are asked to report different reasons for doing their job (“I put effort into my job”). In particular, identified motivation (three items, e.g., “Because I personally consider it important to put effort into this job”) and intrinsic motivation (three items, e.g., “Because the work I do is interesting”) were reported on a scale ranging from 1 (not at all for this reason) to 7 (exactly for this reason).

#### Positive Affect

Positive affect was measured using a short version of the Positive and Negative Affect Scale (Kercher, 1992; Solberg, 2013). The six items (e.g., “I generally feel inspired”) were reported on a scale ranging from 1 (very little) to 5 (very much).

#### Negative Affect

Negative affect was measured using a short version of the Positive and Negative Affect Scale (Kercher, 1992; Solberg, 2013). The six items (e.g., “I generally feel upset”) were reported on a scale ranging from 1 (very little) to 5 (very much).

#### Life Satisfaction

Life satisfaction was measured with five items (e.g., “I am satisfied with my life”) from the Satisfaction with Life Scale (Diener et al., 1985). All items were reported on a scale ranging from 1 (strongly disagree) to 7 (strongly agree).

#### Somatic Symptom Burden

Somatic symptom burden (e.g., “headache”) was measured by the Somatic Symptom Scale-8 (Gierk et al., 2014). The respondents were asked to rate their experience of the eight symptoms (e.g., headaches, dizziness, and back pain) during the previous 4 weeks. Responses were made on a 5-point scale ranging from 1 (not bothered) to 5 (strongly bothered).

#### Emotional Exhaustion

Emotional exhaustion at work was measured using a subscale of the Maslach Burnout Inventory (Maslach et al., 1996) with five items (e.g., “I feel burnt out from my work”) reported on a 7-point scale ranging from 1 (never) to 7 (always).

#### Work Effort

Work effort was assessed using a scale developed by Kuvaas and Dysvik (2009). Five items (e.g., “I try to work as hard as possible”) were reported on a scale ranging from 1 (strongly disagree) to 7 (strongly agree).

#### Work Quality

Work quality was assessed using a scale developed by Kuvaas and Dysvik (2009). Five items (e.g., “The quality of my work is top-notch”) were reported on a scale ranging from 1 (strongly disagree) to 7 (strongly agree).

### Study 2 Participants and Procedures

Participants were 207 employees (55.7% women, 44.3% men) from six knowledge-intensive firms in Norway, with an age range of 20–70 years (*M* = 45.96, *SD* = 11.93). Of the respondents, 73.2% had higher education (bachelor’s degree or higher).

The data were collected using an electronic survey distributed through an e-mail invitation.

### Study 2 Measures

In study 2, the measurement scales for psychological detachment, work motivation, and emotional exhaustion were the same as in Study 1, with the exception of psychological detachment, which was reported on a scale ranging from 1 (completely disagree) to 7 (completely agree). In addition, the following measures were used.

#### Vigor

The vigor subscale of the Utrecht Work Engagement Scale (UWES-17; Schaufeli et al., 2006) assessed vigor at work with six items (e.g., “At my work, I feel bursting with energy”). Responses were made on a scale ranging from 1 (never) to 7 (daily).

#### Work–Home Interference

Work–home interference was assessed with five items (e.g., “Your work comes in conflict with your private life”) developed by Kopelman et al. (1983). The respondents were asked to rate how often they experienced the various statements on a scale ranging from 1 (never) to 7 (always).

#### Sleep Quality

Sleep quality was assessed with the Karolinska Sleep Questionnaire (Kecklund, 1992). The respondents rated how often they had experienced the various difficulties related to sleep in the previous 3 weeks on four items on a scale ranging from 1 (never) to 6 (always).

### Data Analyses

Data analyses were performed using M*plus* (M*plus* version 8.1; Muthén, 2018) in both studies if not otherwise specified. First, confirmatory factor analyses (CFA) were conducted for all study variables. The following goodness-of-fit (GOF) indices were used to evaluate the factor structure for the instruments (Schermelleh-Engel et al., 2003; Brown and Moore, 2012): comparative fit index (CFI) ≥ 0.90, Tucker–Lewis index (TLI) ≥ 0.90, standardized root mean square residual (SRMR) ≤ 0.08, and root mean square error of approximation (RMSEA) ≤ 0.08. After finding acceptable model fit for all latent variables in the CFA, internal consistency was evaluated by score reliability using SPSS Statistics 24 (Cronbach, 1951).

Latent profile analysis (LPA) was used for exploring and identifying different subgroups within the samples based on the participants’ responses to the variables of psychological detachment, identified regulation, and intrinsic motivation (Berlin et al., 2014). The goal of LPA is to classify individuals from a heterogeneous population into smaller, more homogeneous subgroups based on individuals’ values on continuous variables (Berlin et al., 2014). This person-centered approach has the advantage that it groups individuals within a group who are more similar than individuals between groups (Jung and Wickrama, 2008).

The models were specified using the maximum likelihood estimator (MLR). A stepwise comparison of models was conducted when evaluating a one-profile model with successively more profiles (Nylund et al., 2007). In both studies, models with one to four models were tested for a combination of GOF indices and profile sizes (>5%) (Jung and Wickrama, 2008). The GOF indices used in the current study were as follows: the smallest Bayesian information criteria (BIC; Henson et al., 2007) and Akaike’s information criterion (AIC; Akaike, 1987) to assess model fit; the highest possible entropy to assess classification accuracy; posterior probability to asses class separation; a significant *p*-value on the bootstrap likelihood ratio test (BLRT; McLachlan and Peel, 2000); and the Lo–Mendell–Rubin adjusted likelihood ratio test (LMR; Lo et al., 2001). The latter two tests are used to evaluate whether the *k*−1 profile model is rejected in favor of the *k* profile model (Nylund et al., 2007; Jung and Wickrama, 2008; Wickrama et al., 2016). Finally, both theoretical justifications and an evaluation of substantial meaning of profiles were conducted by the researchers when deciding on the number of profiles (Jung and Wickrama, 2008).

To explore for differences between profiles in relation to the associated variables in the study, the automatic three-step BCH approach was used (for detailed description, see Asparouhov and Muthén, 2014). The BCH analysis offers an omnibus test that includes differences between the profiles on each distal outcome variable (Bolck et al., 2004), which is shown to be the most robust and flexible approach yielding the least biased estimates in relation to other comparative analysis exploring differences between profiles (Bakk and Vermunt, 2016). In addition to looking for significant differences in the associated variables between the profiles, the effect size (ES) of the possible differences was explored and interpreted: Cohen’s d ES: 0.01–0.19 (very small), 0.20–0.49 (small), 0.50–0.79 (moderate), 0.80–1.19 (large), 1.20–1.99 (very large), and 2.00 (huge) (Sawilowsky, 2009).

## Results

### Results of the CFA and Internal Consistency – Studies 1 and 2

The results of the CFA indicated an acceptable fit for all variables in both studies (Table 1). It should be noted that the CFA for the two-dimensional constructs of autonomous work motivation (intrinsic regulation and identified regulation), affect (positive and negative affect), and work performance (effort and quality) were, respectively, run simultaneously to confirm the factorial validity of these particular scales. Also, the results of the internal consistency for all variables in the two studies yielded sufficient alpha scores (α; Cronbach, 1951; Table 1). Correlation tables for all study variables in respectively Study 1 and Study 2 can be found in Supplementary Material.

**TABLE 1:** Results of confirmatory factor analysis and internal consistency by alpha for Studies 1 and 2.

**Study**	**Variable**	**χ ^2^(*df*)**	**CFI**	**TLI**	**RMSEA (90% CI)**	**SRMR**	**α**
1	Psychological detachment	1.49(1)*	1.00	0.99	0.05 (0.00–0.19)	0.01	0.80
1	Identified regulation and intrinsic motivation	13.17(8)	0.99	0.98	0.05 (0.00–0.10)	0.03	0.83/0.53
1	Positive affect and negative affect	78.87(51)*	0.98	0.97	0.04 (0.02–0.07)	0.04	0.86/0.83
1	Life satisfaction	5.73(3)	0.99	0.98	0.06 (0.00–0.14)	0.01	0.89
1	Somatic symptom burden	26.66(18)	0.96	0.94	0.05 (0.00–0.08)	0.05	0.72
1	Emotional exhaustion	6.58(4)	0.98	0.97	0.05 (0.00–0.12)	0.02	0.82
1	Work performance: effort and quality	80.00(33)*	0.93	0.90	0.08 (0.06–0.10)	0.05	0.82/0.81
2	Psychological detachment	0.78(1)	1.00	1.00	0.00 (0.00–0.18)	0.01	0.88
2	Identified regulation and intrinsic motivation	12.99(8)	0.99	0.98	0.06 (0.00–0.11)	0.03	0.85/0.96
2	Emotional exhaustion	1.25(3)	1.00	1.00	0.00 (0.00–0.08)	0.01	0.89
2	Vigor	19.08(8)	0.98	0.95	0.08 (0.03–0.13)	0.04	0.88
2	Work–home interference	7.74(4)	0.99	0.98	0.07 (0.00–0.14)	0.03	0.88
2	Sleep quality	3.68(0)*	0.98	1.00	0.00 (0.00–0.00)	0.02	0.86

* < 0.05; χ^2^, chi-squared; df, degrees of freedom.

### Results of the LPA – Studies 1 and 2

The overall results from the stepwise comparison of the number of profiles in studies 1 and 2 favored a two-profile solution (Table 2). Overall, for both studies, the AIC and the BIC decreased until a three-profile solution and increased at a four-profile solution. The results showed significant results on the BLRT and LMR for both studies at a two- and three-profile solution. However, the results for the four-profile solution indicated a non-significant result for study 1 on both the BLRT and LMR, and a non-significant result on the LMR for study 2. The number of participants in each profile at the three-profile solution showed small samples for one of the profiles in both study 1 (*n* = 12, 5%) and study 2 (*n* = 16, 8%). For study 1 at least, such a small profile (5% of total population) is not recommendable (Jung and Wickrama, 2008). Furthermore, the overall findings of the posterior probabilities, along with the result that the entropy was highest at a two-profile solution in both study 1 (0.89) and study 2 (0.85), favored a two-profile solution of the measures of class separation (Wickrama et al., 2016). Finally, the results from both studies indicated that the two-profile solution was best when evaluating substantial meaning and theoretical justifications for the number of profiles (Jung and Wickrama, 2008).

**TABLE 2:** Model fit indices for latent profiles based on detachment, identified regulation, and intrinsic motivation for Studies 1 and 2.

**Study 1**	**Number of profiles**	**Number of free parameters**	**AIC**	**BIC**	**Adjusted BIC**	**BLRT (*p*-value)**	**LMR (*p*-value)**	**Entropy**	**Posterior probability**	**Latent profile proportions (%)**
	1	6	2100.43	2121.28	2102.27				1.00	
	2	10	2018.78	2053.55	2021.55	0.000	0.03	0.89	0.87/0.98	22/217 (9/91)
	3	14	1976.02	2024.69	1980.31	0.000	0.001	0.77	0.84/0.89/0.89	12/113/114 (5/47/48)
	4	18	2149.57	2212.15	2155.10	0.32	0.12	0.78	0.82/0.86/0.96/0.86	108/6/5/120 (44/3/2/50)
**Study 2**	1	6	2083.54	2103.50	2084.49				1.00	
	2	10	1974.93	2008.21	1976.53	0.000	0.002	0.85	0.88/0.96	39/167 (19/81)
	3	14	1937.10	1983.69	1939.33	0.000	0.05	0.76	0.96/0.87/0.90	16/82/108 (8/40/52)
	4	18	1926.11	1986.01	1928.98	0.02	0.18	0.77	0.89/0.92/0.85/0.86	28/12/71/95 (14/6/34/46)

For both studies, the two profiles emerging from the data could be characterized as “Lower involvement employees” (Low-IE) and “Higher involvement employees” (High-IE), due to the profile differentiation on scores on the three variables of psychological detachment, identified regulation, and intrinsic motivation. The Low-IE profile was characterized by higher scores on psychological detachment and lower scores on identified and intrinsic motivation compared with the High-IE profile (Table 3). In study 1, profile Low-IE (*n* = 22) had the following scores on the study variables: detachment (*M* = 3.08, *SD* = 1.59), identified regulation (*M* = 3.43, *SD* = 2.58), and intrinsic motivation (*M* = 3.07, *SD* = 2.77). Profile High-IE (*n* = 217) had the following scores on the study variables: detachment (*M* = 2.63, *SD* = 0.88), identified regulation (*M* = 5.54, *SD* = 1.47), and intrinsic motivation (*M* = 5.36, *SD* = 1.62). In study 2, profile Low-IE (*n* = 39) had the following scores on the study variables: detachment (*M* = 4.67, *SD* = 2.12), identified regulation (*M* = 3.82, *SD* = 1.93), and intrinsic motivation (*M* = 2.92, *SD* = 2.12). Profile High-IE (*n* = 167) had the following scores on the study variables: detachment (*M* = 4.06, *SD* = 1.55), identified regulation (*M* = 5.87, *SD* = 1.10), and intrinsic motivation (*M* = 5.45, *SD* = 1.55).

**TABLE 3:** An overview over the different latent profiles for Studies 1 and 2.

	**Study 1**					**Study 2**				
	**Profile 1, *n* = 22, “Lower involvement”**		**Profile 2, *n* = 217 “Higher involvement”**			**Profile 1, *n* = 39 “Lower involvement”**		**Profile 2, *n* = 167 “Higher involvement”**		
						
**Variable**	***M(SD)***	**CI 95%**	***M(SD)***	**CI 95%**	***d***	***M(SD)***	**CI 95%**	***M(SD)***	** CI 95%**	***d***
Psychological detachment	3.08 (1.59)	[2.41, 3.75]	2.63 (0.88)	[2.51, 2.75]	0.35	4.67 (2.12)	[4.01,5.34]	4.06 (1.55)	[3.83, 4.28]	0.36
Identified regulation	3.43 (2.58)	[2.34, 4.51]	5.54 (1.47)	[5.35, 5.73]	1.01	3.82 (1.93)	[3.21, 4.43]	5.87 (1.10)	[5.69, 6.04]	1.31
Intrinsic motivation	3.07 (2.77)	[1.91, 4.23]	5.36 (1.62)	[5.15, 5.57]	1.01	2.92 (2.12)	[2.27, 3.58]	5.45 (1.55)	[5.22, 5.68]	1.36

**d = Cohen’s d Effect size: 0.01–0.19 (very small), 0.20–0.49 (small), 0.50–0.79 (moderate), 0.80–1.19 (large), 1.20–1.99 (very large), and 2.00 (huge) (Sawilowsky, 2009)*.*

### Differences Between Profiles on Associated Variables – Studies 1 and 2

The results of the BCH analyses examining the differences between the profiles on the associated variables are presented in Table 4. In study 1, the results indicated that the Low-IE profile was significantly lower in the variables of positive affect, life satisfaction, work effort, and quality at work, and significantly higher in exhaustion compared with the High-IE profile. The ES of these differences was small for the variables of effort and quality at work, large for exhaustion, and very large for positive affect and life satisfaction (Sawilowsky, 2009). There were no significant differences between the profiles for the variables somatic symptom burden and negative affect. In study 2, the results indicated that the Low-IE profile was significantly higher in the variables of exhaustion, work–home interference, and sleep quality, and significantly lower in vigor compared with the High-IE profile. The ES of these differences was small for work–home interference, moderate for exhaustion and sleep quality, and large for vigor (Sawilowsky, 2009).

**TABLE 4:** Differences between profiles on distal outcome variables for Studies 1 and 2.

** Study 1**	** Distal outcome variables**	**Profile 1, *n* = 22 “Lower involvement” *M(SD)***	**Profile 2, *n* = 217 “Higher involvement” *M(SD)***	**1 vs. 2 χ ^2^*/p*-value**	**1 vs. 2 Cohen’s *d* ES**
	Positive affect	2.07 (0.98)	3.55 (0.74)	45.24/0.000	1.70
	Negative affect	2.44 (1.03)	2.00 (0.88)	3.60/0.058	0.46
	Life satisfaction	3.47 (2.25)	6.64 (2.06)	39.17/0.000	1.47
	Somatic symptom burden	1.87 (0.75)	1.64 (0.59)	1.90/0.168	0.34
	Emotional exhaustion	3.45 (1.41)	2.29 (0.88)	13.99/0.000	0.99
	Work effort	5.39 (1.17)	6.15 (0.74)	8.61/0.003	0.78
	Work quality	4.75 (0.98)	5.27 (0.74)	5.42/0.02	0.60

**Study 2**	**Distal outcome variables**	**Profile 1, *n* = 39 “Lower involvement” *M(SD)***	**Profile 2, *n* = 167 “Higher involvement” *M(SD)***	**1 vs. 2 χ^2^*/p*-value**	**1 vs. 2 Cohen’s *d* ES**

	Emotional exhaustion	3.88 (1.81)	2.68 (1.29)	14.44/0.000	0.76
	Work–home interference	4.00 (1.75)	3.31 (1.42)	4.85/0.028	0.43
	Vigor	4.60 (1.44)	5.62 (1.03)	16.52/0.000	0.81
	Sleep quality	3.15 (1.25)	2.56 (1.03)	7.21/0.007	0.52

**Cohen’s d effect size: 0.01–0.19 (very small), 0.20–0.49 (small), 0.50–0.79 (moderate), 0.80–1.19 (large), 1.20–1.99 (very large), and 2.00 (huge) (Sawilowsky, 2009)*.*

## Discussion

The purpose of the present study was to examine whether psychological detachment as a job recovery strategy has the same positive benefits for all employees when their quality of work motivation is taken into account. Specifically, we explored whether there are different profiles based on psychological detachment from work and autonomous work motivation, and how these profiles are related to workers’ well-being and work functioning. The results indicated two distinct profiles across two samples of knowledge workers: Low-IE (higher detachment and lower autonomous regulation) and High-IE (lower detachment and higher autonomous regulation). The overall results from the two studies revealed that the High-IE profile was significantly higher on positive affect, life satisfaction, work effort, and work quality, while significantly lower on emotional exhaustion compared with the Low-IE profile. The theoretical and practical implications of these findings are now discussed.

### Theoretical Implications

In previous research, the overall evidence on what predicts well-being among employees at work clearly suggests that both higher levels of autonomous work motivation (Deci et al., 2017) and higher levels of psychological detachment (Sonnentag et al., 2017) are beneficial. These findings are also highlighted in studies using person-centered approaches to examine employees’ motivational profiles at work. Employees with more self-determined profiles of work motivation report higher levels on outcomes such as work engagement, job satisfaction, work performance, and quality of work life, while lower levels on outcomes such as burnout and job anxiety (Howard et al., 2016; Abós et al., 2018; Gillet et al., 2018). However, to our knowledge, no previous study has explored person-centered profiles comprising both work motivation and recovery variables among employees. There is a call to use more person-centered approaches within organizational psychology that allow us to better systematize the data in distinct profiles, both qualitatively and quantitatively (Morin et al., 2018). The present study thereby fills a gap in the current literature by adding nuanced knowledge on motivational profiles with the inclusion of a recovery variable in the analyses.

To some extent, the profiles found in the current study showed unexpected yet interesting theoretical profiles based on previous research. First, employees in the Low-IE profile reported higher levels of psychological detachment and thus have a more adaptive (better) detachment profile, while at the same time reporting lower levels of autonomous work motivation, indicating a more maladaptive (worse) motivational profile. In contrast, the High-IE profile reported lower levels of psychological detachment, indicating a more maladaptive detachment profile, and higher levels of autonomous work motivation and, thus, have a more adaptive motivational profile. These profiles can potentially provide a more nuanced perspective on the implications of detachment related to the question regarding whether some employees benefit more or less from psychological detachment from work (Sonnentag, 2018). It is therefore of interest to explore further the results by looking at the differences between the two profiles regarding the outcomes of psychological well-being and work behavior.

Given that employees in the Low-IE profile reported higher levels of psychological detachment and thus have a better detachment profile, such employees could be expected to gain in terms of work functioning and well-being. However, this profile actually seems worse off by reporting poorer outcomes due to the lower levels of autonomous work motivation. Moreover, High-IE profile employees with lower levels of detachment—arguably negative in terms of workers’ functioning and well-being—are actually better off on both indicators of well-being and work performance. This finding might shed light on the unexpected result of a mixed-method study of elite coaches in football (Bentzen et al., 2017), whereby the unrelated association between coaches’ longitudinal scores of lower levels of psychological detachment and their psychological ill-being was explained by the qualitative finding that the time spent thinking about work during their non-work time was used for problem solving, and thereby experienced as re-energizing. Furthermore, similar conclusions can be found in a study by ten Brummelhuis et al. (2017), who discovered that working long hours was not related to bad health (indexed by risk factors of metabolic syndrome, RMS) and that having a compulsive work mentality (i.e., workaholism) was only detrimental for employees low in engagement. When work engagement was high, workaholism was actually negatively related to RMS. The authors therefor conclude that work engagement may actually protect workaholics from severe health risks. In the same vein, autonomous motivation play an important role for employees’ well-being in relation to lack of psychological detachment. As such, not being able to switch off thinking about work during one’s leisure time might not always lead to unhealthy or negative consequences. Given the profound positive implications of autonomous work motivation on employee well-being and work functioning reported previously (for a review, see Deci et al., 2017), this might not be surprising. It can be argued that autonomously motivated employees think positively about their work in times of non-detachment, thereby not making non-detachment from work something detrimental. In particular, as autonomously motivated employees experience their work as personally meaningful and valuable and/or as something interesting and fun (Gagné et al., 2015), when these employees engage in job-related activities or thoughts during their non-work time, it might not deplete their energy and lead to maladaptive consequences. Rather, when thinking about their work, they experience positive feelings that contribute positively to their well-being. Thus, the results indicate that it is not necessarily a matter of the level of detachment alone, but the combination with the worker’s quality of motivation that determines the outcome. It is important to note that this combination applied to the largest group of workers. Moreover, it is important to note that given the cross-sectional nature of the data, longitudinal studies might reveal detrimental effects of low detachment over time.

In terms of the differences between the profiles related to the outcomes, we see some patterns across the two studies. Specifically, for the indicators of well-being (i.e., positive affect, life satisfaction, and vigor), we detect the strongest and most consistent findings. In contrast, for ill-being (i.e., negative affect, emotional exhaustion, somatic symptom burden, work–home interference, and sleep quality), we see weaker and less consistent findings. These results could stem from the focus on autonomous work motivation in the current study. Past studies have indeed indicated that autonomous work motivation is a better predictor of the bright path of motivational processes, while controlled motivation is more related to the dark path where maladaptive outcomes, such as ill-being, are the result (Trépanier et al., 2015).

Employees in the High-IE profile reported significantly better work effort and work quality. The effort–recovery model argues that psychological detachment is essential for employees to perform optimally at work, and some meta-analytic findings have shown a small positive association between psychological detachment from work and work performance (Wendsche and Lohmann-Haislah, 2017); however, several studies document a negative association between psychological detachment from work during non-work time and indicators of performance (Eschleman et al., 2014; de Bloom et al., 2015; Headrick et al., 2019). The findings of the present study may shed light on these ambiguous results in the literature. In particular, the quality of work motivation might play a role in the nature of this relationship in that detachment alone is not necessarily beneficial for performance as it might take the employee longer to get back into their work after non-work time. Coupled with autonomous work motivation that makes less psychological detachment non-depleting of energy, work performance is enhanced as non-detachment in these states does not take away the employee’s energy. However, it is important to note that work performance was only included in the first study, and that this finding regarding work performance had the lowest ES.

### Practical Implications

In the present research, a differentiated picture of worker autonomous work motivation in combination with psychological detachment provides valuable insights into the nuances of these variables for employee well-being and work functioning. According to the replicated findings of the favorable effects of higher levels of autonomous motivation, organizations should focus on promoting quality motivation among employees. In particular, creating a work environment that is supportive of employees’ basic psychological needs for autonomy, competence, and relatedness by, for instance, taking employees’ perspectives, providing choice and participation, and acknowleging their skills and effort seems essential (Olafsen et al., 2017). However, this is not to say that organizations should not pay attention to job recovery, and, in particular, employees’ psychological detachment from work, as research has highlighted important benefits of this job recovery strategy on employee well-being and work functioning (Sonnentag et al., 2017). Nevertheless, it seems important to be aware of, and individualize, the need for recovery, based on the quality of employee motivation. In times when employees find their work less meaningful and inspirational, it is essential to be more conscious of the need to psychologically detach during non-work time in order to foster employee sustainability, psychological health, and work performance.

### Limitations and Future Research

The current study has several limitations. The data collections in both samples were cross-sectional in nature, with the implication that the results cannot indicate any causal relationships between the study variables (Cook et al., 2002). Even though the findings from the two different samples found conceptually similar results with respect to the aim of the study, the samples within each study could be considered relatively small. A larger sample size could have given clearer results, leading to additional meaningful profiles. Furthermore, a large sample size and longitudinal data could strengthen the findings regarding changes over a longer period of time (e.g., using growth curves; Mäkikangas et al., 2010) or with more frequent measures over time to study daily fluctuations, especially for the variable of psychological detachment (e.g., using diary studies; Derks et al., 2014). As this is the first known study to examine the interplay between autonomous work motivation and psychological detachment, it is, of course, necessary for the study to be replicated with other samples.

## Data Availability Statement

The raw data supporting the conclusions of this article will be made available by the authors, without undue reservation, to any qualified researcher.

## Ethics Statement

The studies involving human participants were reviewed and approved by NSD – Norwegian Centre for Research Data. The patients/participants provided their written informed consent to participate in this study.

## Author Contributions

AO was in charge of the design of the study as well as the data collection. MB was in charge of doing the data analyses of the results. AO and MB cooperated and contributed significantly to the writing of all parts of the manuscript. AO and MB approved the submitted version of the manuscript.

## Conflict of Interest

The authors declare that the research was conducted in the absence of any commercial or financial relationships that could be construed as a potential conflict of interest.
