# Supplementary material for: Benefits of Psychological Detachment From Work: Does Autonomous Work Motivation Play a Role?
Source: Front Psychol. 2020 Apr 30;11:824. doi: 10.3389/fpsyg.2020.00824 (PMC7205444; doi:10.3389/fpsyg.2020.00824)
Supplement: Supplementary file 1 [file Data_Sheet_1.docx]

Supplementary

Table 1

*Correlations Among Variables Study 1*

|  | 1 | 2 | 3 | 4 | 5 | 6 | 7 | 8 | 9 |
| --- | --- | --- | --- | --- | --- | --- | --- | --- | --- |
| 1. Psychological detachment |  |  |  |  |  |  |  |  |  |
| 2. Identified regulation | -.08 |  |  |  |  |  |  |  |  |
| 3. Intrinsic regulation | .01 | .46** |  |  |  |  |  |  |  |
| 4. Positive affect | -.03 | .45** | .15* |  |  |  |  |  |  |
| 5. Negative affect | -.25** | -.10 | -.08 | -.14* |  |  |  |  |  |
| 6. Life satisfaction | .13* | .38** | .15* | .69** | -.61** |  |  |  |  |
| 7. Somatic symptom burden | .002 | -.09 | .-.04 | -.18** | .35** | -.39** |  |  |  |
| 8. Emotional exhaustion | -.15* | -.25** | -.20** | -.27** | .39** | -.40** | .55** |  |  |
| 9. Work effort | -.27* | .36** | .20** | .26** | -.01 | -.21** | -.10 | -.13* |  |
| 10. Work quality | -.05 | .32** | .26** | .31** | -.18** | .35** | -.06 | -.14* | .47** |

*Note*. *N* = 239. *p < .05, two-tailed. **p < .01, two-tailed.

Table 2

*Correlations Among Variables Study 2*

|  | 1 | 2 | 3 | 4 | 5 | 6 |
| --- | --- | --- | --- | --- | --- | --- |
| 1. Psychological detachment |  |  |  |  |  |  |
| 2. Identified regulation | -.09 |  |  |  |  |  |
| 3. Intrinsic regulation | -.16* | .71** |  |  |  |  |
| 4. Emotional exhaustion | -.22* | -.22** | -.35** |  |  |  |
| 5. Work-home interference | -.46** | -.22** | -.17* | .59** |  |  |
| 6. Vigor | .00 | .36** | .56** | -.53** | -.34** |  |
| 7. Sleep quality | -.11 | -.12 | -.20* | .37** | .30** | -.26** |

*Note*. *N* = 207. *p < .05, two-tailed. **p < .01, two-tailed.
